# Direct Ink Writing of SiCN/RuO_2_/TiB_2_ Composite Ceramic Ink for High-Temperature Thin-Film Sensors

**DOI:** 10.3390/ma17153792

**Published:** 2024-08-01

**Authors:** Yusen Wang, Lida Xu, Xiong Zhou, Fuxin Zhao, Jun Liu, Siqi Wang, Daoheng Sun, Qinnan Chen

**Affiliations:** Department of Mechanical and Electrical Engineering, Xiamen University, Xiamen 361102, China13009133326@163.com (S.W.)

**Keywords:** direct ink writing, RuO_2_, high-temperature, thin-film sensor

## Abstract

Direct ink writing (DIW) of high-temperature thin-film sensors holds significant potential for monitoring extreme environments. However, existing high-temperature inks face a trade-off between cost and performance. This study proposes a SiCN/RuO_2_/TiB_2_ composite ceramic ink. The added TiB_2_, after annealing in a high-temperature atmospheric environment, forms B_2_O_3_ glass, which synergizes with the SiO_2_ glass phase formed from the SiCN precursor to effectively encapsulate RuO_2_ particles. This enhances the film’s density and adhesion to the substrate, preventing RuO_2_ volatilization at high temperatures. Additionally, the high conductivity of TiB_2_ improves the film’s overall conductivity. Test results indicate that the SiCN/RuO_2_/TiB_2_ film exhibits high linearity from room temperature to 900 °C, high stability (resistance drift rate of 0.1%/h at 800 °C), and high conductivity (4410 S/m). As a proof of concept, temperature sensors and a heat flux sensor were successfully fabricated on a metallic hemispherical surface. Performance tests in extreme environments using high-power lasers and flame guns verified that the conformal thin-film sensor can accurately measure spherical temperature and heat flux, with a heat flux sensor response time of 53 ms. In conclusion, the SiCN/RuO_2_/TiB_2_ composite ceramic ink developed in this study offers a high-performance and cost-effective solution for high-temperature conformal thin-film sensors in extreme environments.

## 1. Introduction

With the rapid development in the aerospace field, the optimization design and simulation require precise in situ surface condition parameters [1,2]. However, key components often operate in extreme environments characterized by high temperatures, high pressures, high centrifugal forces, and severe erosion. Traditional discrete sensors are no longer adequate for high-precision measurements in the aerospace industry due to their large size and thermal mismatch issues [3,4,5,6]. Thin-/thick-film sensors (TFSs) have emerged as a crucial direction for sensor development in extreme environments because of their small size, high sensitivity, minimal structural damage, and high accuracy [7,8,9,10]. Nevertheless, traditional physical vapor deposition (PVD) methods struggle to achieve uniform and effective thin-film deposition on the complex surfaces of aerospace components. In recent years, direct ink writing technology has emerged as a promising method for conformal deposition of high-temperature thin-film sensors on complex curved surfaces. This method represents a significant advancement in addressing the challenges of thin-film deposition in aerospace applications [11,12,13].

Currently, the ink materials commonly used for high-temperature sensors are predominantly precious metals (such as AgPd, Pt, PtRh) [14,15,16,17], but the high cost of these materials restricts their widespread application. Although ceramic inks [12,18,19] (such as TiB_2_, indium tin oxide (ITO) [20,21], etc.) have been developed, thin-film sensors made from these ceramics exhibit poor linearity, limiting their engineering applications. RuO_2_, known for its excellent electrical conductivity, high-temperature resistance, and high linearity, has been widely utilized in integrated circuits and electronic components. However, RuO_2_ tends to oxidize to gaseous RuO_4_ at high temperatures [22], leading to instability in the electrical performance of the sensors. Current encapsulation materials such as silicate glass or SiO_2_ can enhance the stability of RuO_2_, but these materials often reduce their conductivity [23,24,25]. Hence, there is an urgent need to develop a composite ink that can enhance the high-temperature stability of RuO_2_ without compromising its conductivity. Ceramic precursor polymers can be converted into conductive amorphous ceramics upon high-temperature pyrolysis [26,27,28,29], making them an excellent choice for ink carriers. However, ceramic precursor polymers undergo up to 60% shrinkage during high-temperature pyrolysis [30,31], and thin films made solely from RuO_2_ and ceramic precursors are prone to cracking and detachment. Adding fillers is an effective strategy to prevent thin-film cracking and enhance film adhesion [32]. TiB_2_, as a non-oxide ceramic, generates TiO_2_ and B_2_O_3_ at high temperatures [11], which not only reduces film cracking but also improves the conductivity of the film, making it an optimal material for preparing RuO_2_ composite inks.

In this study, we developed a SiCN precursor and RuO_2_/TiB_2_ composite ink, successfully applying it to the fabrication of thin-film heat flux sensors and resistance thermometers on metallic hemispherical surfaces. This composite ink utilizes the SiCN precursor as the carrier and RuO_2_ as the primary conductive component. The synergistic effect of B_2_O_3_ generated from TiB_2_ and the SiO_2_ glass phase converted from the SiCN precursor enhances film density, encapsulates RuO_2_ to reduce its volatilization, and adjusts the thermal matching of the film layer, thereby improving its adhesion to the substrate. Meanwhile, TiB_2_ also enhances the film’s conductivity. High-temperature tests demonstrated that the SiCN/RuO_2_/TiB_2_ film exhibited excellent high-temperature resistance, with high linearity of resistance from room temperature to 900 °C, good stability (0.1%/h resistance drift at 800 °C), and high conductivity (4410 S/m). As a proof of concept, we printed SiCN/RuO_2_/TiB_2_ ink on a metallic hemispherical surface to create conformal heat flux sensors and resistance thermometers. Extreme environment tests using high-power lasers and butane torches confirmed that the SiCN/RuO_2_/TiB_2_ composite thin-film sensors could accurately monitor the surface temperature and heat flux of metal components.

## 2. Materials and Methods

### 2.1. Materials

The RuO_2_ powder, with an average particle size of 200 nm, was sourced from Suzhou Sub-Nano New Material Co., Ltd., Suzhou, China. High-temperature resistant Ag ink was procured from Chengdu Hehe Micro-Nano Technology Co., Ltd. (Chengdu, China). Titanium diboride (TiB_2_) powder, featuring a particle size of 1 μm, was obtained from Shanghai Chaowei Nanomaterials Co. PSN2 (Shanghai, China) [33], the precursor of SiCN ceramics, was commercially acquired from the Institute of Chemistry, Chinese Academy of Sciences (Beijing, China).

### 2.2. Fabrication Method

**Figure 1:**
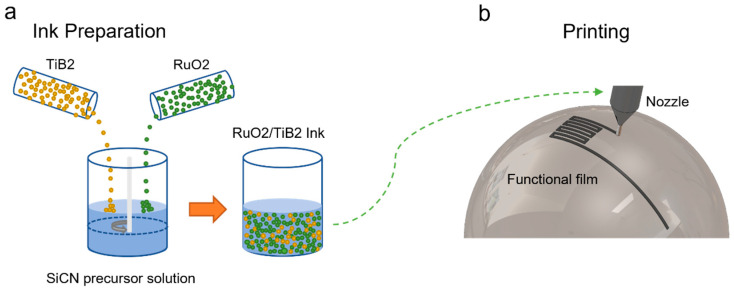
Preparation of SiCN/RuO_2_/TiB_2_ composite ink and sensor manufacturing. (**a**) Ink preparation; (**b**) 3D-printing patterning.

The SiCN/RuO_2_/TiB_2_ composite ink was formulated by thoroughly mixing RuO_2_ micron-scale powder and TiB_2_ micron-scale powder with PSN2 as the organic solvent in a 2:1:1 ratio, utilizing a mixer for one hour (Figure 1a). The SiCN/RuO_2_/TiB_2_ composite ink was loaded onto a Wittenburg technology-based 3D direct writing platform [34] equipped with a 330 μm nozzle. The direct writing platform was used to print the SiCN/RuO_2_/TiB_2_ composite ink on a curved surface. The motor speed of the platform was set to 3000 rpm, with a movement speed of 0.2 mm/s. The microneedle used had a diameter of 160 μm, and the distance between the needle tip and the curved surface was maintained at 5 μm. The pattern to be printed was designed using CAD software(Version T.53.0.0 AutoCAD 2023).

### 2.3. Characterization Methods

Scanning electron microscope (SEM) images were captured using a Zeiss Sigma 300. (Oberkochen, Germany). X-ray diffraction (XRD) analysis was conducted using a Shimadzu XRD-6100 instrument (Kyoto, Japan). Thermoelectric potential measurements were performed using a Keysight 34972A data acquisition system (DAQ)(Santa Rosa, CA, USA) within a tube furnace. Temperature was recorded with a commercially available K-type thermocouple (Taizhou China). An infrared thermal imager, Fluke Ti480 PRO (Everett, WA, USA), capable of measuring temperatures up to 1000 °C, was employed for thermal imaging.

### 2.4. Testing Methods

The temperature-resistance test system comprised a quartz tube furnace (OTF-1200X, MTI KJ GROUP, Hefei China), a data acquisition device (KEYSIGHT 34972A), and a K-type thermocouple (KPS-K, Taizhou China). The K-type thermocouple and SiCN/RuO_2_/TiB_2_ composite resistor were placed together in the constant temperature zone of the tube furnace, with signals from both the thermocouple and resistor being collected simultaneously. The heat flux test system included a data acquisition unit (KEYSIGHT 34972A) and a 1064 nm laser, which was directly aimed at the sensor surface. The laser power was controlled to adjust the heat flux input.

## 3. Results

### 3.1. Sintering and Characteristics of the Conformal High-Temperature Sensitive Layer

**Figure 2:**
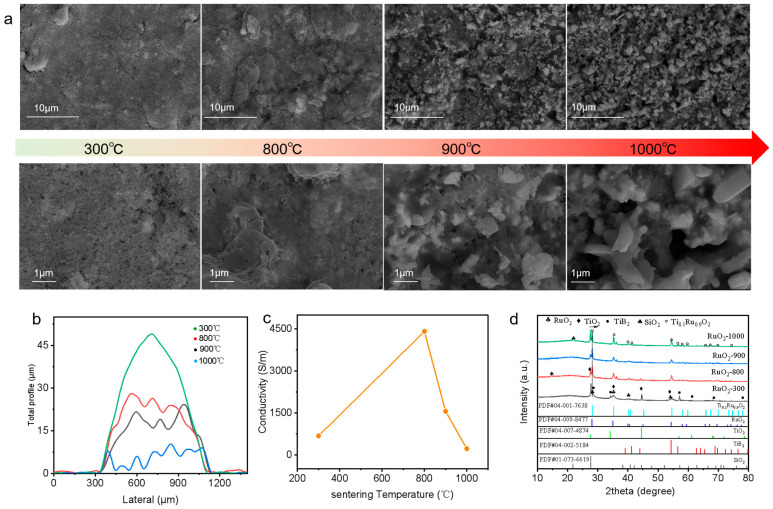
Surface characteristics of the SiCN/RuO_2_/TiB_2_ composite film. (**a**) Surface morphology of the composite film at different sintering temperatures. (**b**) Cross-sectional changes of the film after annealing at different sintering temperatures. (**c**) Graph of conductivity versus sintering temperature. (**d**) XRD pattern.

High-temperature sintering is crucial for the performance of the SiCN/RuO_2_/TiB_2_ composite ink under elevated temperatures. Figure 2a analyzes the morphological changes of the composite film after sintering at different temperatures. After holding at 300 °C for 1 h, no significant sintering was observed, and the film thickness, measured by a profilometer, was approximately 49 μm. TiB_2_ began to oxidize to TiO_2_. At around 400 °C, TiB_2_ oxidation produced B_2_O_3_ [11]. The XRD images did not show the presence of B_2_O_3_ because it is amorphous. After sintering at 800 °C for 1 h, significant changes were observed on the film surface. The SiCN underwent a glass transition, forming SiO_2_ with insulating properties at high temperatures. Together with the TiO_2_ and B_2_O_3_ produced from the oxidation of TiB_2_, these oxides encapsulated the RuO_2_ particles, preventing their oxidation to volatile RuO_4_. At this stage, the film shrank longitudinally by approximately 50% (Figure 2b), resulting in a more compact structure and the formation of conductive pathways within the film. The addition of TiB2 increased the film’s conductivity to 4410 S/m (Figure 2c), which is higher than previously reported values [23]. We also tested the performance of the SiCN/RuO_2_ composite ink. We found that the conductivity of the film sintered from the SiCN/RuO_2_ composite ink was lower compared to the SiCN/RuO_2_/TiB_2_ composite film (Figures S1 and S2). Additionally, the SiCN/RuO_2_ film exhibited higher porosity and lower density. After sintering at 900 °C, more cracks appeared on the composite film surface, and RuO_2_ particles began to be exposed. The film thickness further decreased, likely due to the volatilization of some SiO_2_-B_2_O_3_-TiO_2_ ternary oxides at high temperatures, allowing some oxygen to diffuse into the film at high temperatures [35], leading to a reduction in conductivity to approximately 1555 S/m. After sintering at 1000 °C for 1 h, the SiO_2_, B_2_O_3_, and other oxides continued to volatilize, exposing more RuO_2_ particles on the film surface. Some RuO_2_ particles volatilized at high temperatures, reducing the film thickness to only 7.5 μm. The conductivity decreased to 219.8 S/m, and XRD analysis indicated the formation of significant amounts of Ti_0.1_Ru_0.9_O_2_ in the sample sintered at 1000 °C.

**Figure 3:**
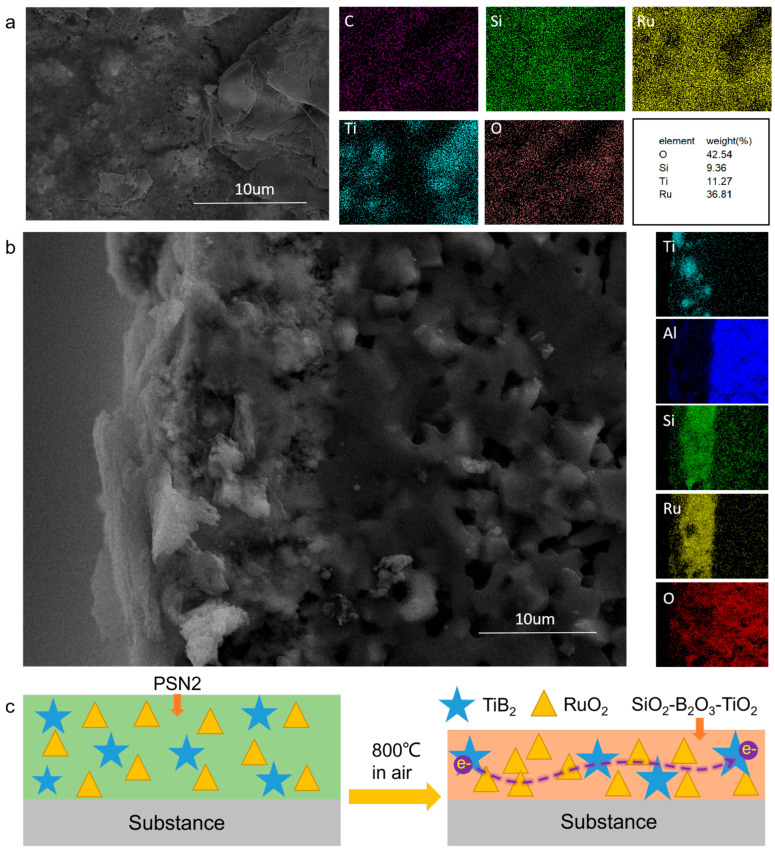
(**a**)Surface morphology and EDS elemental mapping of the printed SiCN/RuO_2_/TiB_2_ composite film. (**b**) Cross-sectional surface morphology and EDS elemental mapping of the film sintered at 800 °C for 1 h. (**c**) Sintering process schematic diagram.

The EDS results (Figure 3) corroborated these observations. After sintering the composite ink at 800 °C for 1 h, Si, Ru, and O were evenly distributed on the substrate surface, with O being the most abundant, while Ti and Si were present in the lowest proportions. By EDS analysis, it can be seen that the distribution of Ti elements is non-continuous, while Ru elements are uniformly and continuously distributed in the film. This indirectly indicates that the conductive pathways are formed by the interconnected RuO_2_ particles, and the randomly distributed TiB_2_, due to its good conductivity, plays a role in reducing electron mobility resistance. This is equivalent to increasing the cross-sectional area of the conductive paths, thus enhancing conductivity. The distribution characteristic of O elements suggests that SiO_2_, generated during the ceramicization of SiCN at high temperatures, coordinated with B_2_O_3_ and TiO_2_ generated by TiB_2_ to encapsulate the RuO_2_ particles, thereby preventing their volatilization in high-temperature environments. Consistent with the planar results, the cross-sectional EDS analysis revealed that Ru and O had the highest content, while Ti was the least abundant. The tendency of Ti particles to increase in size at high temperatures was evident. The boundary between the composite film and the alumina substrate in the EDS analysis was visible, exhibiting a continuous interface without pores, indicating excellent film quality. The composite film demonstrated good thermal expansion matching performance. These results indicate that the ternary thermally grown oxide of SiO_2_-B_2_O_3_-TiO_2_, generated from the SiCN material and TiB_2_ at high temperatures, enhances the overall performance of the SiCN/RuO_2_/TiB_2_ composite film. As clearly shown in Figure 3c, during the sintering process of the SiCN/RuO_2_/TiB_2_ composite ink at 800 °C, PSN2 decomposes at high temperatures to form SiO_2_. This SiO_2_, along with B2O_3_ and TiO_2_ produced from the partial oxidation of TiB_2_, encapsulates the RuO2 particles, preventing O_2_ from penetrating the interior of the film. Concurrently, the film becomes thinner and denser, which facilitates the formation of conductive pathways between RuO_2_ and TiB_2_, thereby enhancing the electrical conductivity.

### 3.2. Testing the Thermistor Performance of SiCN/RuO_2_/TiB_2_ Composite Ink Film

**Figure 4:**
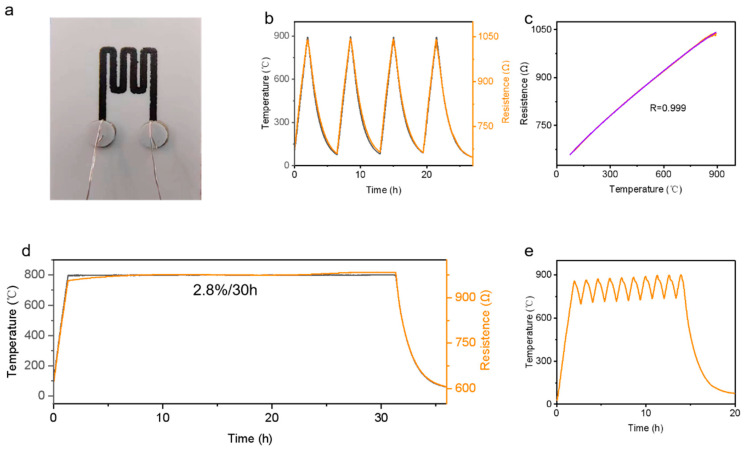
Thermistor performance testing of the SiCN/RuO_2_/TiB_2_ composite film thermistor. (**a**) Test object image. (**b**) Cold and hot cycle test. (**c**) Second-order fitting of experimental data. (**d**) Test results after 30 h of holding at 800 °C. (**e**) Durability test under high-temperature cyclic conditions.

The ceramicization of the SiCN precursor at high temperatures, along with the production of B_2_O_3_ and TiO_2_ from TiB_2_, collectively contributes to filling the gaps among RuO_2_ particles. This increases the film density, forms conductive paths, and ensures stable conductivity at high temperatures. Figure 4a depicts samples fabricated by 3D printing technology, where SiCN/RuO_2_/TiB_2_ composite ink is deposited onto aluminum oxide plates for temperature testing purposes. Testing the composite ink film sensor over four thermal cycles from room temperature (30 °C) to 900 °C revealed that the thermistor curve between 30 °C and 900 °C demonstrated a high fit with an R^2^ value of 0.999 (Figure 4c), indicating excellent high-temperature resistance for the composite film. Few materials [16,36,37,38,39] have been able to maintain such a good linear relationship over such a wide temperature range, making this composite a potential substitute for precious metals in high-temperature sensor applications.

To verify its durability at high temperatures, a resistivity change curve at 800 °C for over 30 h was measured (Figure 4d), showing a resistance drift rate of only 0.1%/h at 800 °C. This confirms the exceptional high-temperature stability of the composite film, surpassing the stability of previously reported materials. The output characteristics of the film’s thermistor under alternating temperatures between 700 °C and 900 °C for over 10 h were also measured. The output results were nearly identical to the temperature signal reflected by the type-K thermocouple, confirming the composite material’s excellent stability at high temperatures (Figure 4e).

### 3.3. Application Verification of the SiCN/RuO_2_/TiB_2_ Composite Ink Conformal Thin-Film Thermistor Array

**Figure 5:**
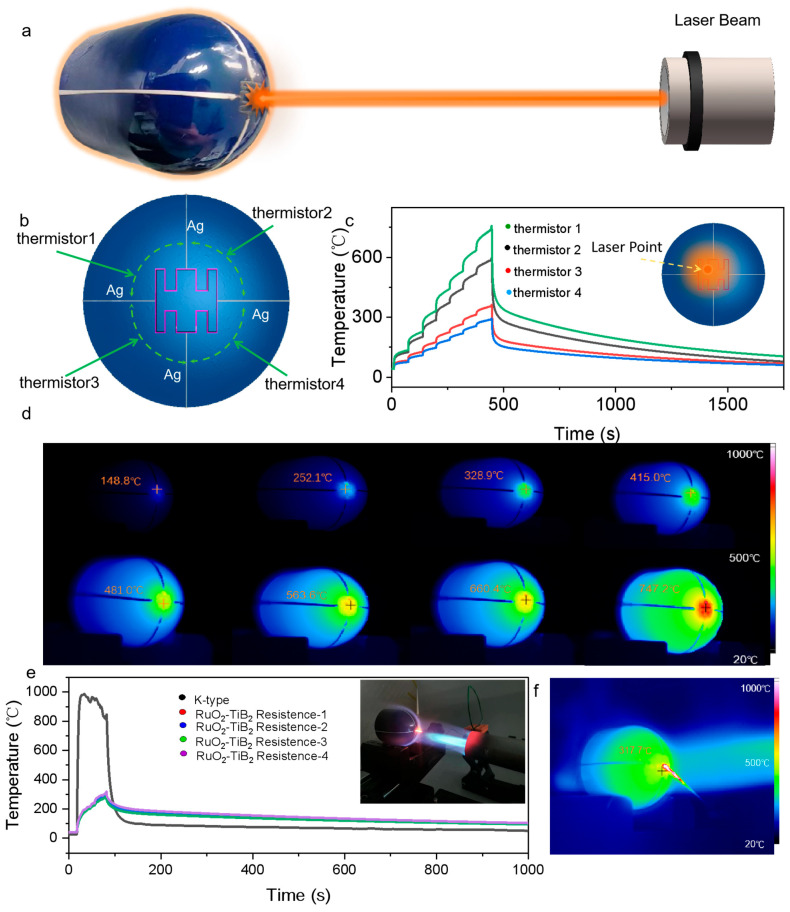
Harsh environment testing of the SiCN/RuO_2_/TiB_2_ composite material thermistor array. (**a**) Schematic of laser irradiation on the metallic hemispherical surface thermistor array. (**b**) Thermistor array design scheme. (**c**) Test results of gradually increasing laser power on a single thermistor on the metallic hemispherical surface. (**d**) Infrared thermal imaging at 60s under a certain power of laser irradiation. (**e**) Comparison of conformal film thermistor and K-type thermocouple under the flame gun impact. (**f**) Infrared thermal imaging under flame gun impact.

To further illustrate the composite material’s distributed temperature monitoring characteristics on complex surfaces under extreme conditions, lasers and flame guns were employed to simulate such environments. A 1064 nm fiber continuous laser was used to directly irradiate a thermistor located in the upper left corner of the thermistor array, with the power incrementally increased by 15 W every 60 s, up to a maximum of 105 W (Figure 5a). Simultaneously, the infrared thermal imager records the temperature data at the 60th second after each power change (Figure 5d). The resistance of four thermistors was recorded in real time using four conformal Ag film wires, and the fitting formula from Figure 4c was used to calculate the temperature of the measured sensitive layer. The temperature calculated from the thermistor directly irradiated by the laser was 757.75 °C, closely matching the 747.2 °C displayed by the infrared thermal imager. The other three thermistors displayed temperatures of 582.14 °C, 362.29 °C, and 287.87 °C, respectively, with temperature differences influenced by the distance between the thermistor and the laser’s direct spot.

To demonstrate the temperature difference caused by a high-temperature gas flow impact between using a discrete thermocouple and a conformal thermistor array, a K-type thermocouple was affixed to the sensitive layer. A butane flame torch was aimed at the middle position of the spherical sensitive layer while simultaneously collecting data from the thermistor and the K-type thermocouple. As shown in Figure 5e, the K-type thermocouple reading instantly rose to a high temperature of 980 °C, while the maximum temperature calculated from the thermistor was only 319 °C. The K-type thermocouple has a low thermal mass itself, so it instantly rises above 900 °C when affected by the flame impact. However, the metallic hemisphere has a larger thermal mass, causing it to heat up more slowly. The surface temperature was only about 319 °C, while the infrared thermal imager displayed a metallic hemisphere surface temperature of approximately 317.7 °C. The temperature measurement results of the conformal film sensor are closer to the actual temperature of the surface of the structural component.

Traditional discrete temperature sensors often require modification of the original structure for installation, disrupting the consistency and continuity of heat conduction in the component. This results in severe “ cold/hot spot effects “ during the measurement process, causing the measured temperature to often not reflect the true temperature of the component. However, a conformal film temperature sensor can avoid this problem due to its micrometer-level thickness and in situ manufacturing method, thereby achieving precise in situ temperature monitoring.

Experimental proofs show that under the two extreme conditions of laser and flame gun exposure, the SiCN/RuO_2_/TiB_2_ composite ink conformal thin-film heat-sensitive resistor exhibits excellent high-temperature stability. This verifies that the composite material has the potential to serve as a new raw material for high-temperature sensors.

### 3.4. Application Test of the Conformal Film Heat Flux Sensor

**Figure 6:**
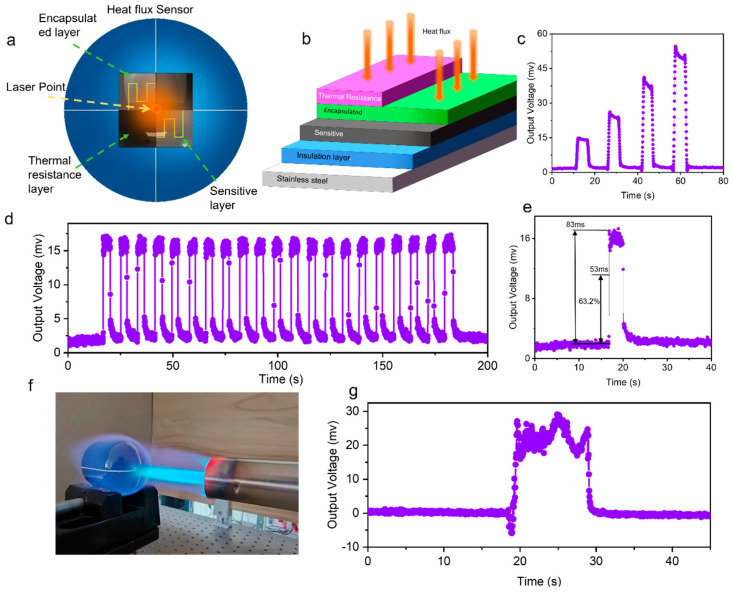
(**a**) Schematic of laser irradiation on the metallic hemispherical surface heat flux sensor. (**b**) Structural principle schematic of the heat flux sensor. (**c**) Test results under different heat fluxes. (**d**) Test results of 21 cycles under the same heat flux. (**e**) Response time test results. (**f**) Optical image during flame gun impact test. (**g**) Test results of the heat flux sensor under flame gun impact.

Measuring temperature alone cannot adequately reflect the dynamic process of heating or cooling of components under extreme conditions. Heat flux measurement makes the heat transfer process more evident and accurate [40,41]. The sensitive layer pattern designed in this work, based on the Wheatstone bridge principle, can function as a SiCN/RuO_2_/TiB_2_ composite ink temperature sensing array or as a conformal film heat flux sensor. Figure 6b illustrates the structural characteristics of the heat flux sensor, which comprises the metal substrate, electrical insulation coating, sensitive layer, packaging layer, and thermal resistance layer. When the heat flux sensor receives heat flow, the different resistances in the sensitive layer’s four resistors, caused by the thermal resistance layer’s thickness, create a differential output.

The sensitive layer is connected to a direct current voltage, converting resistance changes into output voltage changes in the circuit. The heat flow signal is provided by a 1064 nm fiber laser (Figure 6a). To improve the conformal sensor’s surface absorption rate, the surface is typically made black, as the encapsulated layer and thermal resistance layer are made of transparent glaze, affecting the laser absorption rate. This setup yields experimental data, as shown in Figure 6c,d, where gradually increasing the laser power results in a progressively increasing output voltage signal. A continuous heat flux measurement over 21 cycles with 5 s intervals demonstrated the sensor’s excellent high-temperature durability and stability. The response time of the conformal heat flux sensor, made of SiCN/RuO_2_/TiB_2_ composite material, is remarkably fast at only 53 ms, verifying the sensor’s broad application prospects. Furthermore, a rapid impact was applied to the center of the metallic hemispherical surface using a butane flame torch (Figure 6f), resulting in a significant heat flow signal (Figure 6g). This testing reaffirms the potential of the SiCN/RuO_2_/TiB_2_ composite ink as a robust material for high-temperature sensors.

## 4. Conclusions

In summary, this study addresses the volatility and instability of RuO_2_ at high temperatures by proposing a SiCN/RuO_2_/TiB_2_ composite ink with stable performance in high-temperature environments. The composite ink achieves a denser and more adherent high-quality film by filling the pores between RuO_2_ particles with SiO_2_-B_2_O_3_-TiO_2_ ternary thermally grown oxides generated from the SiCN precursor and TiB_2_ at high temperatures. These glass phases also reduce the sintering temperature of RuO_2_, thereby minimizing its volatility under high-temperature conditions. Additionally, the incorporation of TiB_2_ enhances the film’s conductivity (4410 S/m) and prevents film delamination and cracking. Furthermore, thermal sensitivity tests on the SiCN/RuO_2_/TiB_2_ composite film thermistors in high-temperature environments reveal that the resistance-temperature fitting curve exhibits a high correlation (R^2^ = 0.999) from room temperature to 900 °C. The film can operate stably for over 30 h at 800 °C, with a resistance drift rate of only 0.1% per hour, demonstrating the composite film’s potential for engineering applications. To verify the technical feasibility of applying this composite film to conformal film sensors, high-power laser and butane flame torch tests were conducted on a conformal sensor array on the metallic hemispherical surface for extreme environment evaluations of thermistor and heat flux performance. The results indicate that the thermistor array can accurately measure the temperature at different points on the metallic hemispherical surface, showing significant advantages over discrete sensors. The heat flux sensor exhibits a response time of only 53 ms and demonstrates excellent high-temperature durability and stability. Through these tests, the SiCN/RuO_2_/TiB_2_ composite film has met the high standards required for high-temperature conformal sensor applications. The tests on the metallic hemispherical surface highlight the composite material’s broad prospects for in situ measurements in extreme environments.

## Data Availability

The original contributions presented in the study are included in the article/Supplementary Materials, further inquiries can be directed to the corresponding authors.

## Supplementary Materials

The following supporting information can be downloaded at: https://www.mdpi.com/article/10.3390/ma17153792/s1, Figure S1: (a) Graph of the Electrical Conductivity of SiCN/RuO_2_/TiB_2_ Films as a Function of Sintering Temperature (b) Graph of the Electrical Conductivity of RuO_2_/SiCN Films as a Function of Sintering Temperature; Figure S2: SEM image of RuO_2_/SiCN composite ink sintered at 800 °C for one hour; Video S1: printing of the sensitive layer; Video S2: printing of silver conductor; Video S3: Simulation of harsh environment test.
