# Supplementary material for: Direct Ink Writing of SiCN/RuO2/TiB2 Composite Ceramic Ink for High-Temperature Thin-Film Sensors
Source: Materials (Basel). 2024 Aug 1;17(15):3792. doi: 10.3390/ma17153792 (PMC11312801; doi:10.3390/ma17153792)
Supplement: Supplementary file 1 [file materials-17-03792-s001.zip › Figures S1 and S2.pdf]

Supporting Information

Direct Ink Writing of $SiCN/RuO_2/TiB_2$ Composite Ceramic Ink for High-Temperature Thin-Film Sensors

Yusen Wang †, Lida Xu *,†, Xiong Zhou, Fuxin Zhao, Jun Liu, Siqi Wang, Daoheng Sun * and Qinnan Chen *

Department of Mechanical and Electrical Engineering, Xiamen University, Xiamen 361102, China
* Correspondence: superldxu@gmail.com (L.X.); sundh@xmu.edu.cn (D.S.); chenqinnan@xmu.edu.cn (Q.C.)
† These authors contributed equally to this work.

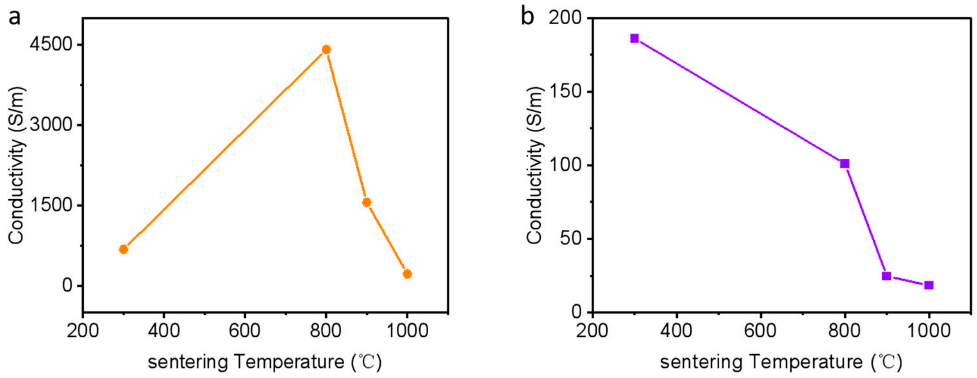

Figure S1 (a) Graph of the Electrical Conductivity of $SiCN/RuO_2/TiB_2$ Films as a Function of Sintering Temperature (b) Graph of the Electrical Conductivity of $RuO_2/SiCN$ Films as a Function of Sintering Temperature

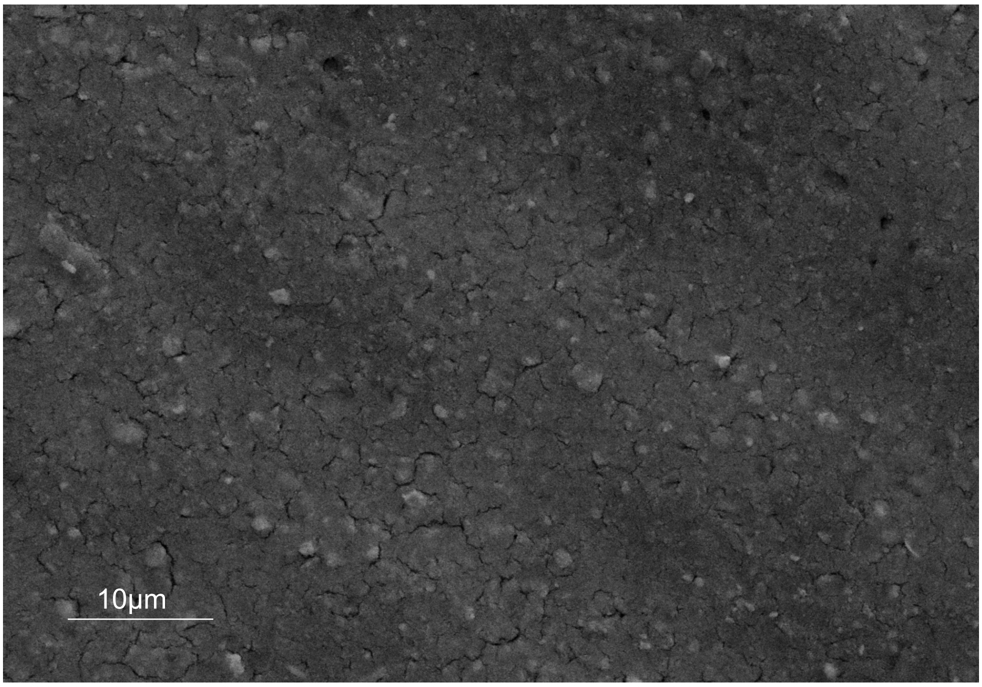

Figure S2 SEM image of $RuO_2/SiCN$ composite ink sintered at 800°C for one hour
